# Selection of the optimal channel configuration for implementing wearable EEG devices for the diagnosis of mild cognitive impairment

**DOI:** 10.1186/s13195-022-01115-3

**Published:** 2022-11-12

**Authors:** Kyeonggu Lee, Kang-Min Choi, Seonghun Park, Seung-Hwan Lee, Chang-Hwan Im

**Affiliations:** 1 School of Electronic Engineering, Hanyang University, Seoul, Republic of Korea; 2 Department of Psychiatry, Inje University Ilsan Paik Hospital, Goyang, Republic of Korea; 3 Clinical Emotion and Cognition Research Laboratory, Inje University Ilsan Paik Hospital, Goyang, Republic of Korea; 4 School of Biomedical Engineering, Hanyang University, Seoul, Republic of Korea

**Keywords:** Mild cognitive impairment, Electroencephalography, Wearable EEG device, Machine learning, Optimal channel configuration

## Abstract

**Background:**

Early diagnosis of mild cognitive impairment (MCI) is essential for timely treatment planning. With recent advances in the wearable technology, interest has increasingly shifted toward computer-aided self-diagnosis of MCI using wearable electroencephalography (EEG) devices in daily life. However, no study so far has investigated the optimal electrode configurations for the efficient diagnosis of MCI while considering the design factors of wearable EEG devices. In this study, we aimed to determine the optimal channel configurations of wearable EEG devices for the computer-aided diagnosis of MCI.

**Method:**

We employed an EEG dataset collected from 21 patients with MCI and 21 healthy control subjects. After evaluating the classification accuracies for all possible electrode configurations for the two-, four-, six-, and eight-electrode conditions using a support vector machine, the optimal electrode configurations that provide the highest diagnostic accuracy were suggested for each electrode condition.

**Results:**

The highest classification accuracies of 74.04% ± 4.82, 82.43% ± 6.14, 86.28% ± 2.81, and 86.85% ± 4.97 were achieved for the optimal two-, four-, six-, and eight-electrode configurations, respectively, which demonstrated the possibility of precise machine-learning-based diagnosis of MCI with a limited number of EEG electrodes. Additionally, further simulations with the EEG dataset revealed that the optimal electrode configurations had significantly higher classification accuracies than commercial EEG devices with the same number of electrodes, which suggested the importance of electrode configuration optimization for wearable EEG devices based on clinical EEG datasets.

**Conclusions:**

This study highlighted that the optimization of the electrode configuration, assuming the wearable EEG devices can potentially be utilized for daily life monitoring of MCI, is necessary to enhance the performance and portability.

**Supplementary Information:**

The online version contains supplementary material available at 10.1186/s13195-022-01115-3.

## Introduction

Mild cognitive impairment (MCI) is a psychiatric syndrome characterized by a cognitive decline greater than that expected based on an individual’s age and education level [1]. MCI is believed to be associated with several underlying causes, especially for Alzheimer’s disease (AD) [2]. As approximately 40–60% of patients with MCI have an underlying AD pathology [3, 4], early detection of MCI is important for postponing cognitive decline and preventing its conversion to AD [5]. In general, however, a patient’s visit to the hospital for the diagnosis of MCI is considerably delayed because MCI does not usually interfere with daily activities, and individual symptoms are heterogeneous depending on the etiology and cognitive reserve [6, 7]. Moreover, conventional methods for diagnosing MCI such as history recording, clinical questionnaires, and simple cognitive tests are susceptible to misdiagnosis particularly in the early stages of MCI [8]. The exploration of all cognitive domains and quantification of overall cognitive performance are essential for avoiding potential diagnostic errors. However, such a time-consuming and tedious diagnostic procedure might act as a barrier preventing the early detection of MCI by discouraging potential patients from visiting a medical clinic [7].

Various biomarkers have been suggested for a more quantitative and objective diagnosis of MCI such as those extracted from cerebrospinal fluid (CSF), magnetic resonance imaging (MRI), and electroencephalography (EEG) [7, 9–11]. Recently, biomarkers from EEG have drawn increased attention owing to their advantages of relatively shorter diagnostic time and higher cost-effectiveness [12–15]. The key characteristics of the EEG data of elderly patients with MCI compared with those of normal elderly people (without MCI) are reduced complexity, decrease in inter-regional synchronizations, and shifts in the power spectrum from high-frequency components (alpha, beta, and gamma bands) toward low-frequency components (delta and theta bands) [16, 17]. These EEG biomarkers have been widely employed for the computer-aided diagnosis of MCI based on machine learning (ML) algorithms, thus demonstrating the possibility of a reliably high-performance EEG-based diagnosis of MCI [17–21]. For example, Morabito et al. [20] achieved an accuracy of 85% in classifying MCI and healthy control subjects (HCs) using a convolutional neural network (CNN) model. Fiscon et al. [21] achieved a classification accuracy of 92% for the detection of MCI using a decision tree classifier with wavelet coefficients from the EEG data.

However, previous studies on machine learning-based MCI diagnoses with EEG have generally employed research-grade EEG devices that require the aid of a trained experimenter so that the wider utilization of EEG-based MCI diagnosis in places other than laboratory or clinical environments is prevented [22–24]. Recent developments in wearable EEG technology have made EEG devices portable and inexpensive, thereby expanding their application fields [24–26]. For example, wearable EEG devices have the potential to be used for the early detection of neurological diseases [27, 28]. With the recent developments of hydrogel electrode technologies, it is expected that wearable EEG devices would be widely employed for continuous, long-term EEG monitoring and daily-life diagnosis of neurological diseases [29]. In this regard, these devices could be effective tools in the primary screening of MCI, for which early diagnosis is generally difficult due to the delayed visit to the hospital.

To increase the effectiveness of wearable EEG devices, it is necessary to increase the accessibility of the patients by making the devices portable and affordable [30]. Although attempts have been made to reduce the number of EEG channels while preserving the performance of the devices [31] and to investigate the optimal channel configurations that maximize the performance in brain-computer interfaces and biometric systems [32, 33], to the best of our knowledge, no study was performed to investigate the optimal electrode configurations for wearable EEG devices to maximize the accuracy of the machine learning-based diagnosis of MCI.

In this paper, we present a procedure to determine the optimal electrode configurations for wearable EEG devices to diagnose MCI, considering both the practicality and classification accuracy of wearable EEG devices. We suggest optimal channel configurations for two-, four-, six-, and eight-electrode conditions using a clinical EEG dataset collected from patients with MCI and healthy individuals. Furthermore, we demonstrate that the proposed optimal electrode configurations exhibit statistically higher performance in the diagnosis of MCI than commercial wearable EEG devices.

## Methods and materials

### Determination of optimal electrode configuration

To determine the optimal electrode configurations for different numbers of electrodes, we only considered configurations consisting of electrode pairs positioned symmetrically with respect to the midline (e.g., F7–F8 and FC5–FC6) and electrodes located on the midline (e.g., Fz and Pz), because it was thought that wearable EEG devices with bilateral symmetry are much easier to implement than configurations with randomly distributed electrodes [27]. In other words, the optimal electrode configurations were determined by comparing the classification accuracies of all possible electrode configurations that can be formed with the combinations of midline electrodes (Fz, Cz, Pz, and Oz) and symmetric electrode pairs (Fp1–Fp2, AF3–AF4, F7–F8, F3–F4, FC5–FC6, FC1–FC2, T7–T8, C3–C4, CP5–CP6, CP1–CP2, P7–P8, P3–P4, PO3–PO4, and O1–O2) when the number of electrodes was set to two, four, six, and eight. The number of all possible electrode combinations for the two-, four-, six-, and eight-electrode configurations were 20, 176, 924, and 3276, respectively.

**Fig. 1:**
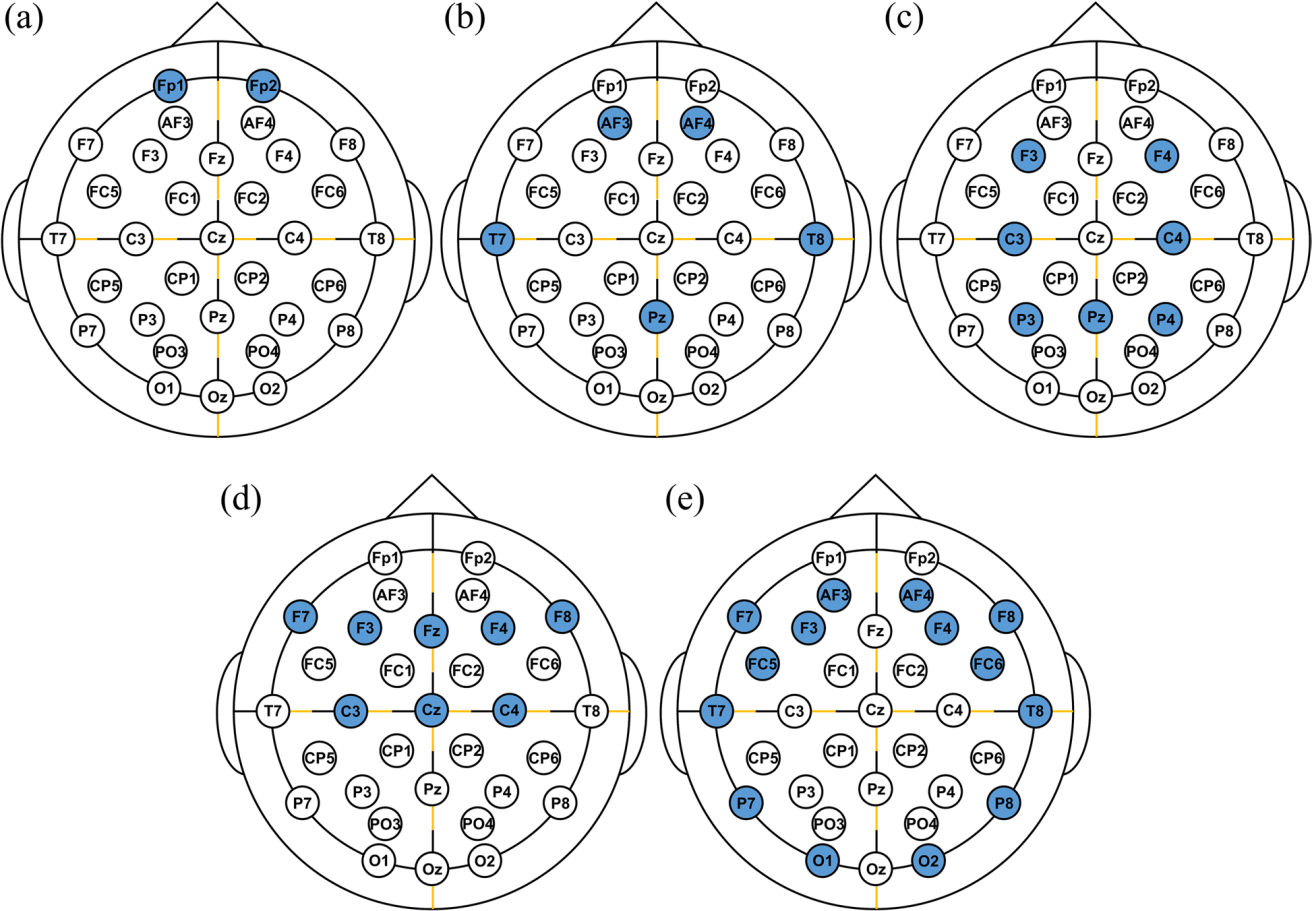
Electrode configurations of commercial wearable EEG devices: **a** Focusband^TM^ (2ch), **b** Insight^TM^ (4ch), **c** DSI-7^TM^ (7ch), **d** Imec^TM^ (8ch), and **e** EPOC^TM^ (14ch)

The optimal electrode configuration was determined by selecting the electrode configuration that exhibited the highest accuracy among all possible channel combinations. After determining the optimal electrode configurations with two, four, six, and eight electrodes, the classification accuracies of the optimal electrode configurations were compared with those of commercial wearable EEG devices including Focusband^TM^ (T2 Green Pty Ltd.; Carrara, QLD, Australia), Insight^TM^ (Emotiv Inc.; San Francisco, CA, USA), DSI-7^TM^ (Wearable Sensing LLC; San Diego, CA, USA), Imec^TM^ (Imec Inc.; Leuven, FB, Belgium), and EPOC^TM^ (Emotiv Inc.; San Francisco, CA, USA). The electrode configurations of commercial devices are shown in Fig. 1.

### Participants

EEG data were acquired from 42 participants (21 patients with MCI and 21 healthy controls [HCs]) at the Inje University Ilsan Paik Hospital. The diagnosis of MCI was based on a clinical evaluation by trained psychiatrists who used the Structured Clinical Interview for DSM-IV (or V) Axis I Disorders (SCID-I) or the Mini International Neuropsychiatric Interview (MINI). None of the patients who participated in the study were pregnant or had any of other neurological or comorbid disorders, organic brain damage, or impairments in sensory or motor functions.

A total of 21 healthy participants were recruited from the local community. They did not satisfy the DSM-IV or V-based lifetime diagnostic criteria for any major psychiatric disorder as screened by the SCID-I Non-Patient Edition (SCID-NP) or MINI-based diagnostic criteria.

Demographic information, including sex, age, and education, was compared between the patients with MCI and HCs. A measurement of educational level is based on the duration of formal education. There were no statistically significant differences between the two groups in terms of demographic characteristics, including sex, age, and education. The detailed demographic information is presented in Table 1. All participants signed a written informed consent form approved by the Inje University Ilsan Paik Hospital Institutional Review Board (IRB no. 2018-12-012-013).

**Table 1:** Demographic information of the MCI and HC groups

	MCI	HC
**N**	21	21
Male / Female	7/14	6/15
*p*	0.7385	
**Age**		
Mean	74.71	73.71
(SD)	(6.50)	(4.63)
*p*	0.5686	
**Education**		
Mean	7.86	9.33
(SD)	(4.42)	(5.47)
*p*	0.3476	

### Signal acquisition and preprocessing

The EEG signal was acquired at a rate of 1000 Hz using a SynAmps amplifier (Neuroscan, Compumedics USA, Charlotte, NC, USA) from 32 scalp electrodes (Fp1, Fp2, AF3, AF4, Fz, F3, F4, F7, F8, FC1, FC2, FC5, FC6, Cz, C3, C4, T7, T8, CP1, CP2, CP5, CP6, Pz, P3, P4, P7, P8, PO3, PO4, Oz, O1, and O2) according to the modified international 10–20 system. The impedance of each electrode was maintained below 5 kΩ throughout the experimental period. Ground and reference electrodes were placed on the forehead and mastoids.

The resting EEG was recorded for 4 min with eyes closed. The acquired EEG data were manually inspected to eliminate blocks contaminated by environmental or physiological noise. Then the EEG data were baseline-corrected by subtracting the average value for each channel and band-pass filtered at cut-off frequencies of 0.5 Hz and 50 Hz using a 6th-order zero-phase Butterworth infinite impulse response filter. Thereafter, the preprocessed resting EEG data were segmented into 5-s epochs without an overlap. We rejected epochs in which the maximal absolute potential value exceeded the threshold of 75 μV; therefore, the numbers of remaining epochs were different among subjects, with twenty being the minimal number of epochs. To equalize the number of epochs for each subject, twenty epochs were randomly selected for each subject.

### Feature extraction

The absolute power spectrum density (APSD), relative power spectrum density (RPSD), differential asymmetry (DASM), rational asymmetry (RASM), phase-amplitude coupling (PAC), Shannon entropy (SE), Hjorth parameters (HP), Lyapunov exponent (LE), Hurst exponent (HE), and Kolmogorov complexity (KC) were extracted as candidate features for MCI diagnosis. These candidate features have been used as biomarkers in previous studies on EEG-based diagnosis of MCI or Alzheimer’s disease (AD) [17, 34–37]. The equations used to compute these features are summarized in Table 2, and their derivation process and detailed description are provided in the Supplementary Material.

**Table 2:** List of EEG features evaluated in this study

Feature	Mathematical expression
**Absolute power spectrum density (APSD)**^a^	$\frac{1}{N}\sum\limits_{n=1}^Nx(n){e}^{-i2\pi fn/N}$
**Relative power spectrum density (RPSD)**	Absolute PSD of specific band/absolute PSD of total band
**Differential asymmetry (DASM) **[32]	Difference between absolute PSDs of inter-hemispheric electrode pairs
**Rational asymmetry (RASM) **[32]	Ratio between absolute PSDs of inter-hemispheric electrode pairs
**Phase-amplitude coupling (PAC)**^b ^[38]	${\textrm{coherence}}_{fph}\left({X}_{ph},{\overset{\sim }{A}}_{ph}\right)$
**Shannon entropy (SE) **[39]	$-\sum\limits_{i=1}^Np\left({x}_i\right)\ln p\left({x}_i\right),\textrm{where}\sum\limits_{i=1}^Np\left({x}_i\right)=1$
**Hjorth parameters (HP)**^c ^[40]	$\textbf{Activity}\left(\boldsymbol{x}\right)=\frac{1}{N}\sum\limits_{i=1}^N{\left({x}_i-{\mu}_i\right)}^2$ $\textbf{Mobility}\left(\boldsymbol{x}\right)=\sqrt{\frac{\upsigma \left({\textrm{x}}^{\prime}\right)}{\upsigma \left(\textrm{x}\right)}}$ $\textbf{Complexity}\left(\boldsymbol{x}\right)=\frac{\textrm{Mobility}\left({\textrm{x}}^{\prime}\right)}{\textrm{Mobility}\left(\textrm{x}\right)}$
**Lyapunov exponent (LE)**^d ^[41]	$\lambda (i)=\frac{1}{i\Delta t}\frac{1}{K}\sum\limits_{\textrm{j}=1}^K\ln \frac{d_j(i)}{d_j(0)}$
**Hurst exponent (HE)**^e ^[42]	log(*R*/*S*)/ log(*N*)
**Kolmogorov complexity**^f^**(KC) **[43]	*c*(*n*)/*b*(*n*)

*x* represents the EEG time-series data and ^a^*N* indicates the length of the data. ^b^ The coherence here is the coherence at frequency *fph* between the time-varying energy of the high-frequency signal (${\overset{\sim }{A}}_{ph}$) and the unfiltered raw signal believed to contain the modulating frequency (*X*_*ph*_), ^c^*μ*_*i*_ represents the mean of *x*, *x*^′^ represents the derivative of *x*, and *σ*(*x*) represents the standard deviation of *x*. ^d^ ∆*t* is the sampling period of the EEG time series, *K* is the embedding dimension, *d*_*j*_(0) is the initial distance from the *j*th point to its nearest neighbor, and *d*_*j*_(*i*) is the distance between the *j*th pair of nearest neighbors after *i* discrete time steps. ^e^*N* is the length of the data sample, *R* is the difference between the maximum deviation from the mean and the minimum deviation from the mean, and *S* is the standard deviation. ^f^*n* is the length of the time-series data, *c*(*n*) reflects the relative complexity of the data, and *b*(*n*) is the ratio between n and log(*n*). The details are described in the Supplementary Material

Spectral features were calculated for each of the following nine sub-frequency bands: delta (δ, 1–4 Hz), theta (θ, 4–8 Hz), low alpha (α_L_, 8–10 Hz), high alpha (α_H_, 10–12 Hz), total alpha (α, 8–12 Hz), low beta (β_L_, 12–18 Hz), high beta (β_H_, 18–30 Hz), total beta (β, 12–30 Hz), and gamma (β, 30–50 Hz).

For the PAC, which evaluates the coherence between the low-frequency phase and high-frequency amplitude, the low-frequency signal was set as either δ or θ, while the high-frequency signal was set as one of the other seven sub-frequency bands (except δ or θ). The total number of candidate features extracted from the 32 channels was 1500 consisting of {9 (APSD) + 9 (RPSD) + 14 (PAC) + 3 (HP) + 1 (SE) + 1 (LE) + 1 (HE) + 1(KC)} × 32(channels) + {9 (DASM) + 9 (RASM)} × 14 (pairs). Each candidate feature was averaged over all twenty epochs per channel, resulting in 1500 averaged candidate features per participant.

### Feature selection and classification

A support vector machine (SVM) classifier was employed using the statistics and machine learning toolbox in MATLAB 2018b (MathWorks, Natick, MA, USA). Leave-pair-out (LPO) cross-validation (CV) was conducted to evaluate the classification accuracy of the model [44]. In detail, the data of two participants (one patient with MCI and one from the HC group) were used as the test set, and the data of the remaining 40 participants (20 patients with MCI and 20 HCs) were used as the training set. Accuracy, sensitivity, and specificity were calculated and averaged over all possible combinations of the two participants (one MCI and one HC; 21 × 21 = 441).

In each iteration of LPO-CV, *z*-score normalization was applied to each feature of the training set [45]:

1 $$z=\frac{X-\overline{X}}{\sigma },$$

where $\overline{X}$ and *σ* denote mean and standard deviation, respectively. The features of the test set were normalized using (1) with the $\overline{X}$ and *σ* calculated from the training set. The optimal feature subset was then selected from the training set based on the rank order according to Fisher’s score, which is one of the most widely used filter methods for supervised feature selection [46]. The feature selection was performed with the candidate features extracted from the target electrode configuration. The maximum number of selected features was set to 15 to avoid potential overfitting. The “*N*-feature accuracy” was evaluated by averaging the results of all LPO-CV iterations for *N* features, where *N* represents the number of features used for the classification, which ranged from 1 to 15. The classification accuracy for each electrode configuration was determined as the highest accuracy among the “*N*-feature accuracies.” Note that the highest accuracy was achieved when the number of features was less than 10 in most cases.

### Statistical analysis

Statistical analysis was conducted to investigate the differences in demographic information between the MCI and HC groups and differences in the classification accuracies between the proposed optimal electrode configurations and those of commercial wearable EEG devices. The chi-square test [47] was conducted to test the difference in sex composition between the MCI and HC groups. A two-tailed Student’s *t*-test [48] was conducted to identify the differences in age and education between the MCI and HC groups as the normality of the data was confirmed by the one-sample Kolmogorov–Smirnov test [49]. In addition, the Bonferroni-corrected Wilcoxon signed-rank test [50, 51] was conducted to identify the difference between the classification accuracies for the proposed electrode configurations and those of the commercial wearable EEG devices as the normality of the data was not confirmed by the one-sample Kolmogorov–Smirnov test.

## Results

**Fig. 2:**
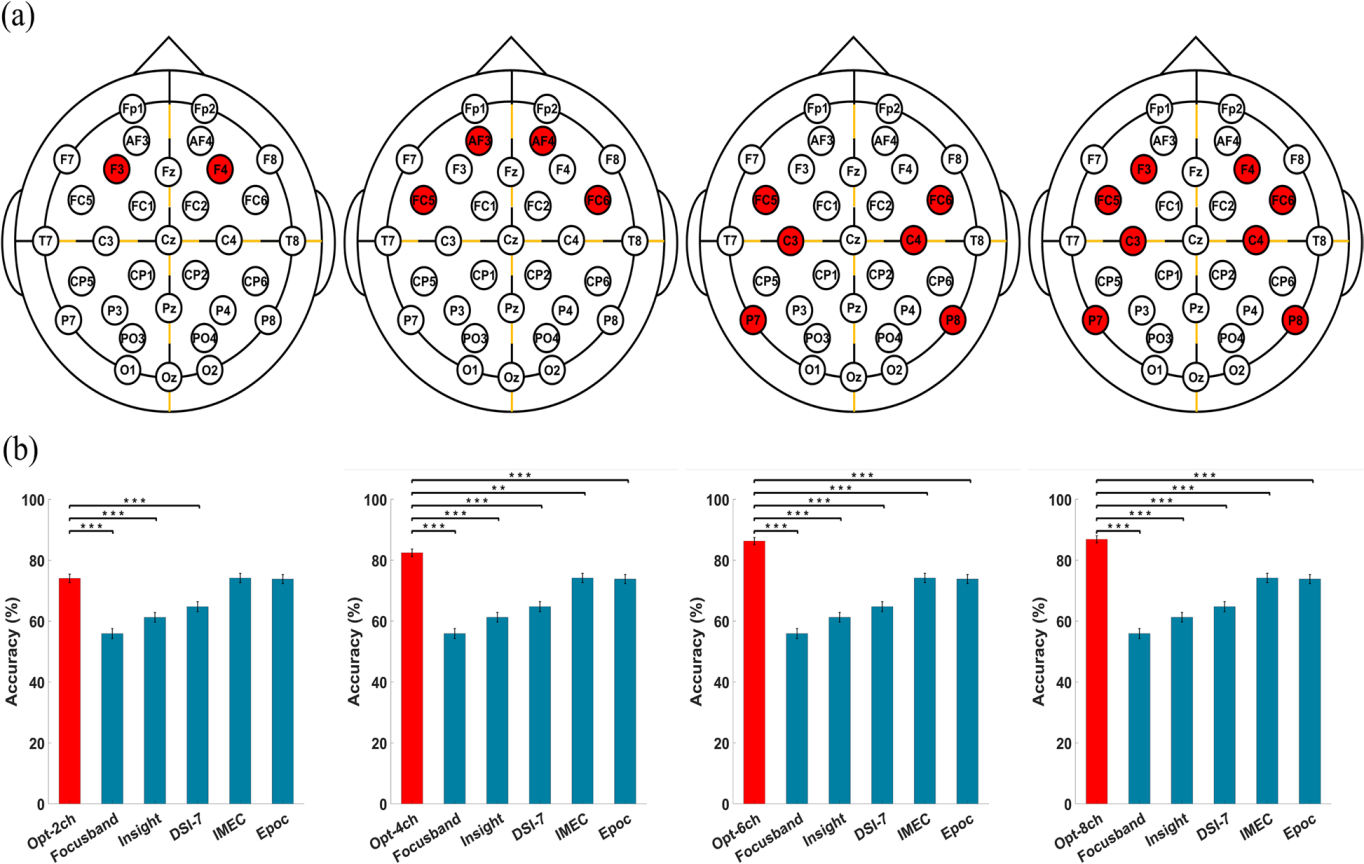
**a** Proposed optimal electrode configurations consisting of two, four, six, and eight channels (from top to bottom). **b** Performance comparison between the proposed optimal electrode configurations and the electrode configurations of commercial wearable EEG devices. The *x*-axis of the bar graphs represents the electrode configurations. The error bars indicate standard error. * Bonferroni-corrected *p* < 0.05, ** Bonferroni-corrected *p* < 0.001, *** Bonferroni-corrected *p* < 0.0001

The optimal electrode configurations that resulted in the highest classification accuracy among all possible channel combinations are presented in Table 3 and Fig. 2a. The optimal two-channel (hereafter denoted by Opt-2ch), four-channel (Opt-4ch), six-channel (Opt-6ch), and eight-channel (Opt-8ch) electrode configurations were “F3–F4,” “AF3–AF4–FC5–FC6,” “FC5–FC6–C3–C4–P7–P8,” and “F3–F4–FC5–FC6–C3–C4–P7–P8,” respectively. All optimal electrode configurations were composed of combinations of the following five electrode pairs: AF3–AF4, F3–F4, FC5–FC6, C3–C4, and P7–P8, which were in the frontal and parietal areas. Notably, Opt-8ch was a combination of Opt-2ch and Opt-6ch, and no midline electrode was included in the optimal electrode configuration.

**Table 3:** Channel combination and diagnostic performances of the optimal electrode configurations and those of five commercial wearable EEG devices

	Accuracy (Mean±SD)	Sensitivity (Mean±SD)	Specificity (Mean±SD)	Channel combination
**Opt-2ch**	74.04% ±4.82	63.95% ±9.23	84.13% ±7.87	F3–F4
**Opt-4ch**	82.43% ±6.14	70.75% ±11.98	94.10% ±4.23	AF3–AF4–FC5–FC6
**Opt-6ch**	86.28% ±2.81	86.17% ±6.55	86.39% ±5.28	FC5–FC6–C3–C4–P7–P8
**Opt-8ch**	86.85% ±4.97	86.85% ±6.88	86.85% ±7.81	F3–F4–FC5–FC6–C3–C4–P7–P8
**Focusband**^**TM**^**(2ch)**	55.90% ±7.01	50.11% ±12.01	61.68% ±8.71	Fp1–Fp2
**Insight**^**TM**^**(5ch)**	61.22% ±7.97	55.10% ±13.58	67.35% ±9.07	Pz–AF3–AF4–T7–T8
**DSI-7**^**TM**^**(7ch)**	64.74% ±7.17	65.76% ±11.72	63.72% ±8.84	Pz–F3–F4–C3–C4–P3–P4
**Imec**^**TM**^**(8ch)**	74.15% ±6.87	70.29% ±10.85	78.00% ±9.58	Fz–Cz–F7–F8–F3–F4–C3–C4
**Epoc**^**TM**^**(14ch)**	73.81% ±7.57	60.77% ±12.41	86.85% ±7.21	AF3–AF4–F7–F8–F3–F4–FC5–FC6–T7–T8–P7–P8–O1–O2

LPO-CV can be regarded as the 21 × 21-fold cross-validation. Therefore, the results of 441 iterations of LPO-CV were first divided into 21 blocks, and then the standard deviation (SD) was calculated across the 21 blocks

The accuracy, sensitivity, and specificity of the machine learning-based MCI diagnosis using optimal electrode configurations are summarized in Table 3. The numbers of features that resulted in the highest accuracies were 2, 2, 6, and 7 for Opt-2ch, Opt-4ch, Opt-6ch, and Opt-8ch, respectively. Accuracies of 74.04% ± 4.82, 82.43% ± 6.14, 86.28% ± 2.81, and 86.85% ± 4.97 were achieved for Opt-2ch, Opt-4ch, Opt-6ch, and Opt-8ch, respectively, which demonstrated the possibility of precise machine learning-based diagnosis of MCI with a limited number of EEG electrodes. It can be seen from the table that the classification accuracy gradually increases as the number of electrodes increases; however, the increment in the accuracy is nearly saturated when the number of electrodes becomes six. Furthermore, while the sensitivity was approximately 20% lower than the specificity for Opt-2ch and Opt-4ch, the difference between the sensitivity and specificity was reduced to less than 1% for Opt-6ch and Opt-8ch.

The accuracy, sensitivity, and specificity of the machine learning-based MCI diagnosis using the electrode configurations of the five commercial wearable EEG devices are provided in Table 3. A statistical comparison of the classification accuracies between the four optimal electrode configurations and the five wearable EEG devices is shown in Fig. 2b. Opt-2ch statistically outperformed the electrode configurations of three commercial devices (Focusband^TM^, Insight^TM^, and DSI-7^TM^, Bonferroni-corrected *p* < 0.001) that were composed of two, five, and seven electrodes, respectively. Opt-2ch did not show significant improvement compared to Imec^TM^ (Bonferroni-corrected *p* = 0.94) and EPOC^TM^ (Bonferroni-corrected *p* = 0.88), which are composed of eight and 14 electrodes, respectively. The other optimal electrode configurations (Opt-4ch, Opt-6ch, and Opt-8ch) statistically outperformed the electrode configurations of all the commercial wearable EEG devices considered in this study (Bonferroni-corrected *p* < 0.001 in all cases).

**Fig. 3:**
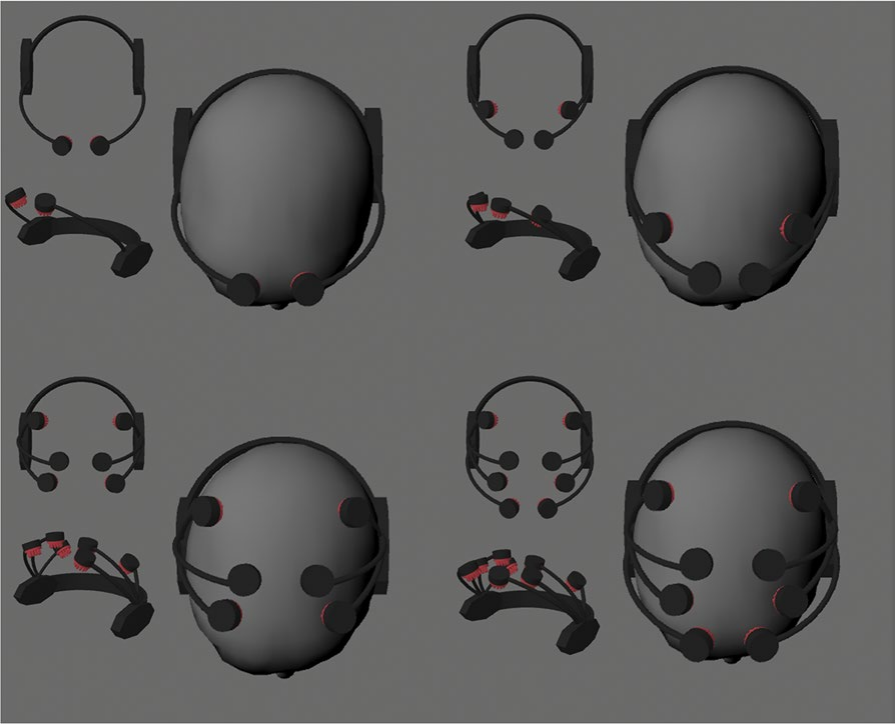
3D-rendered images of wearable EEG devices with the electrode configurations proposed in this study

Figure 3 shows the 3D-rendered concept design of wearable EEG devices with the proposed optimal electrode configuration. 3D images were rendered using Maya 2022 (Autodesk Inc., San Rafael, CA, USA). The overall design concept was inspired by EPOC^TM^ (Emotiv Inc.; San Francisco, CA, USA). In addition, the devices were assumed to have fingered EEG electrodes, which reflected the latest trend in electrode design for wearable EEG devices [24].

## Discussion

In this study, we attempted to determine the optimal electrode configurations that could lead to the highest accuracy in the machine-learning-based diagnosis of MCI. The electrode configurations investigated in this study were composed of a small number of electrodes (two, four, six, and eight electrodes), assuming wearable EEG devices can potentially be utilized for daily life monitoring of MCI. The proposed optimal electrode configurations showed statistically higher accuracies than the electrode configurations of commercial wearable EEG devices, albeit with a smaller number of electrodes. For example, Opt-4ch showed a higher diagnostic accuracy than the electrode configurations of all five commercial wearable EEG devices tested in this study, which highlighted the importance of optimizing the electrode configurations in the design of wearable EEG devices considering the specific purpose of the wearable EEG devices.

We investigated the most frequently selected features in the LPO-CV iterations to identify the EEG features that contributed the most to distinguishing between patients with MCI and HCs. Table 4 shows the four most frequently selected features and their mean values for the MCI and HC groups. The four features were δ DASM between FC5 and FC6, the HE in F3, the HE in C4, and PAC (θ-β_H_) in P8, which were selected at least 397 times out of 441 LPO-CV iterations (approximately 90% of all LPO-CV iterations) for Opt-8ch (see Supplementary Fig. 1). These EEG features have been found to be closely associated with the pathophysiology of MCI in previous studies.

**Table 4:** Comparisons of the most frequently selected feature values between the MCI and HC groups

Feature list	MCI (mean ***±*** SD)	HC (mean ***±*** SD)	***p***-value
**DASM (*δ***) **between FC5 and FC6 (μV**^**2**^**)**	0.4687 ± 0.4653	0.0953 ± 0.1045	** 0.0016
**HE in F3**	0.7806 ± 0.0664	0.7288 ± 0.0505	** 0.0063
**PAC (θ-β**_**H**_**) in P8**	0.1172 ± 0.0100	0.1281 ± 0.0141	* 0.025
**HE in C4**	0.7464 ± 0.0624	0.6948 ± 0.0582	** 0.0054

Statistical analysis was performed using a two-tailed Student’s *t*-test, as the normality of each compared feature was confirmed using the one-sample Kolmogorov–Smirnov test. * *p* < 0.05, ** *p* < 0.01, *** *p* < 0.001

First, the value of δDASM between FC5 and FC6 in patients with MCI was statistically higher than that in the HCs. This result is in line with a previous study that reported that patients with MCI exhibited a significantly higher delta-band power in the left central area than HCs; however, this was not the case for the right central area [52]. Additionally, the HE in F3 and C4 was significantly higher in the MCI group than in the HC group in our study. These results are also in good agreement with those of previous studies. According to John et al*.* [53], the HE of patients with MCI or AD was higher than that of the HCs in whole-brain areas when the HE values were calculated from eyes-closed resting EEG data, as in our study. Lastly, the PAC (θ-β_H_) at P8 was lower in patients with MCI than in the HCs in our study. This result is also consistent with that of Poza et al. [54] reported that patients with mild AD exhibited lower PAC values in the posterior area than HCs.

Although the proposed optimal electrode configurations exhibited statistically higher performance than the electrode configurations of commercial wearable EEG devices, their sensitivities were reported to be approximately 20% lower than the specificities in cases of Opt-2ch and Opt-4ch. In general, well-balanced sensitivity and specificity values are important in designing diagnostic systems. However, as wearable EEG devices are expected to be employed as tools for the primary screening of MCI, an EEG device with higher diagnostic sensitivity is required. Therefore, these optimal electrode configurations could be alternatively determined by selecting the electrode combinations with the highest sensitivity with the overall accuracy maintained at an appropriate level. For example, an electrode pair “C3–C4” showed a classification accuracy of 69.16% ± 7.55, which is approximately 5%p lower than that of Opt-2ch; however, it showed a relatively higher sensitivity of 74.60% ± 11.30 and a moderate level of specificity (63.72% ± 9.58) (see Supplementary Table 1). In the case of the 4-channel configuration, “F3–F4–FC5–FC6” showed an approximately 8% higher sensitivity than Opt-4ch when the difference in accuracy between the two configurations was only 2.5%p (see Supplementary Table 2). If sensitivity is regarded as a more important factor than specificity in designing wearable EEG devices for the primary screening of MCI, the “C3–C4” and “F3–F4–FC5–FC6” configurations might be considered possible alternatives to Opt-2ch and Opt-4ch, respectively. The top 20 electrode configurations for six- and eight-electrode cases can also be found in Supplementary Tables 3 and 4.

The optimal electrode configurations for the diagnosis of MCI may vary depending on several factors, such as the participants and recording devices. Because most EEG features have large inter-subject and/or inter-device variability [55], an additional EEG dataset acquired from a larger number of participants using various EEG devices might be necessary in future studies to further generalize the proposed electrode configurations. It is expected that the optimal electrode configurations will be fine-tuned based on an additional EEG dataset. Additionally, we are planning to manufacture a wearable EEG device with the proposed optimal electrode configuration and investigate in a future study whether the device is effective enough to be used for the early screening of MCI.

## Supplementary Information

**Additional file 1: Supplementary Table 1.** Performances of the top 10 electrode configurations composed of two electrodes. The table lists the calculated accuracies, sensitivities, and specificities for the top 10 electrode configurations with the highest accuracies. These values were used as performance measures. **Supplementary Table 2.** Performances of the top 10 electrode configurations composed of four electrodes. The table lists the calculated accuracies, sensitivities, and specificities for the top 10 electrode configurations with the highest accuracies. These values were used as performance measures. **Supplementary Table 3.** Performances of the top 20 electrode configurations composed of six electrodes. The table lists the calculated accuracies, sensitivities, and specificities for the top 20 electrode configurations with the highest accuracies. These values were used as performance measures. **Supplementary Table 4.** Performances of the top 20 electrode configurations composed of eight electrodes. The table lists the calculated accuracies, sensitivities, and specificities for the top 20 electrode configurations with the highest accuracies. These values were used as performance measures. **Supplementary Figure 1.** List of the most frequently selected features. The features were most frequently selected in all CV iterations for the number of features that yielded the highest accuracy in each optimal electrode configuration. From top to bottom, the feature list of the Opt-2ch, Opt-4ch, Opt6ch, and Opt-8ch configurations are described. The x-axis is “oftenness,” which is calculated by dividing the feature selection times by all CV iteration times (441).

## Data Availability

The data used in this study cannot be open to the public according to the Institutional Review Board approval.
