# Supplementary material for: Selection of the optimal channel configuration for implementing wearable EEG devices for the diagnosis of mild cognitive impairment
Source: Alzheimers Res Ther. 2022 Nov 12;14:170. doi: 10.1186/s13195-022-01115-3 (PMC9652885; doi:10.1186/s13195-022-01115-3)
Supplement: Supplementary file 1 — Additional file 1: Supplementary Table 1. Performances of the top 10 electrode configurations composed of two electrodes. The table lists the calculated accuracies, sensitivities, and specificities for the top 10 electrode configurations with the highest accuracies. These values were used as performance measures. Supplementary Table 2. Performances of the top 10 electrode configurations composed of four electrodes. The table lists the calculated accuracies, sensitivities, and specificities for the top 10 electrode configurations with the highest accuracies. These values were used as performance measures. Supplementary Table 3. Performances of the top 20 electrode configurations composed of six electrodes. The table lists the calculated accuracies, sensitivities, and specificities for the top 20 electrode configurations with the highest accuracies. These values were used as performance measures. Supplementary Table 4. Performances of the top 20 electrode configurations composed of eight electrodes. The table lists the calculated accuracies, sensitivities, and specificities for the top 20 electrode configurations with the highest accuracies. These values were used as performance measures. Supplementary Figure 1. List of the most frequently selected features. The features were most frequently selected in all CV iterations for the number of features that yielded the highest accuracy in each optimal electrode configuration. From top to bottom, the feature list of the Opt-2ch, Opt-4ch, Opt6ch, and Opt-8ch configurations are described. The x-axis is “oftenness,” which is calculated by dividing the feature selection times by all CV iteration times (441). [file 13195_2022_1115_MOESM1_ESM.pdf]

Supplementary Material

1. Feature extraction

The features were calculated for each preprocessed epoch. Each feature of 20 epochs in the channel was averaged. Therefore, each feature had a value per channel.

1.1 Spectral Features

Spectral features were extracted for each of the following nine sub-frequency bands:

- Delta (δ, 1–4 Hz),
- theta (θ, 4–8 Hz),
- low alpha ($L\alpha$, 8–10 Hz),
- high alpha ($H\alpha$, 10–12 Hz),
- alpha (α, 8–12 Hz),
- low beta ($L\beta$, 12–18 Hz),
- high beta ($H\beta$, 18–30 Hz),
- total beta (β, 12–30 Hz), and
- gamma (γ, 30–50 Hz).

First, the absolute power spectral density (APSD) was calculated using the periodogram function in MATLAB 2018b. A rectangular window was used for the calculations. The relative power spectrum density (RPSD), differential asymmetry (DASM), and rational asymmetry (RASM) were calculated

using the APSD value. The feature-associated asymmetry was calculated for each pair of channels (ex, Fp1-Fp2, AF3-AF4, and F3-F4, etc.). The formula for RPSD, DASM, and RASM in a specific sub-band (x band) is

$$RPSD_{x\ band} = \frac{APSD_{x\ band}}{APSD_{total\ band}} \tag{1}$$

$$DASM_{x\ band} = |APSD_{x\ band}\ of\ channel\ A - APSD_{x\ band}\ of\ channel\ B| \tag{2}$$

$$RASM_{x\ band} = \frac{APSD_{x\ band}\ of\ channel\ A}{APSD_{x\ band}\ of\ channel\ B} \tag{3}$$

where channels A and B have symmetrical relationships. Thus, when all 32 channels are used, the total number of EEG spectral features is 9 (APSD) $\times$ 32 (channels) + 9 (RPSD) $\times$ 32 (channels) + 9 (DASM) $\times$ 14 (pairs) + 9 (RASM) $\times$ 14 (pairs) = 828.

1.2 Phase-amplitude coupling (PAC) [1, 2]

PAC indicates the coherence between a low-frequency signal and the time course of power at higher frequencies. We set a low-frequency signal as one of δ and θ and set a high-frequency signal as one of the seven sub-frequencies (excluding δ and θ). Let signal $\{X_t\}$ be represented by time series X_1, X_2, …, X_N. The time course of powers $S_1(f_2)$, $S_2(f_2)$, …, $S_N(f_2)$ was estimated for a higher frequency f_2 by applying a sliding tapered time window followed by a Fourier transformation. The coherency $coherence(f_1, f_2)$ was estimated between the signal $\{X_t\}$ and the time course of the power $\{S_t(f_2)\}$ for a given low-frequency f_1. The coherency was calculated with respect to two time series divided into M segments with L long data points.

$$coherence(f_1, f_2) = \frac{\sum_{k=1}^{M} \tilde{X}^k(f_1)\tilde{S}^{*k}(f_1, f_2)}{\sqrt{\sum_{k=1}^{M} \left|\tilde{X}^k(f_1)\right|^2 \sum_{k=1}^{M} \left|\tilde{S}^k(f_1, f_2)\right|^2}} \tag{4}$$

where

$$\tilde{X}^k(f_1) = \Delta t \sum_{l=1}^{M} h_l X_{L\left(k-\frac{1}{2}\right)+l} e^{-i2\pi f_1 l\Delta t} \; and$$

$$\tilde{S}^k(f_1, f_2) = \Delta t \sum_{l=1}^{M} h_l S_{L\left(k-\frac{1}{2}\right)+l}(f_2) e^{-i2\pi f_1 l\Delta t} \; , h \; is \; hamming \; taper.$$

Coherence, which is the absolute value of coherency, was extracted as a feature. Thus, in each channel, the number of PAC features was 14, which is equal to the number of combinations of $\{f_1, f_2\}$.

1.3 Shannon entropy (SE) [3]

Entropy is a nonlinear measure that quantifies the degree of complexity of a time series. SE is defined as follows:

$$SE = -\sum_{i=1}^{N} p(x_i) \ln p(x_i) \tag{5}$$

where x represents the EEG time-series data, N is the length of the data sample, and $p(x)$ is the probability of x satisfying

$$\sum_{i=1}^{N} p(x_i) = 1 \tag{6}$$

1.4 Hjorth parameters (HP) [4]

The HP is a statistical property of a signal in the time domain. It includes three types of parameters: activity, mobility, and complexity. The parameters are defined as follows:

$$\boldsymbol{Activity}(\boldsymbol{x}) = \frac{1}{N}\sum_{i=1}^{N} (x_i - \mu_i)^2 \tag{7}$$

$$\boldsymbol{Mobility}(\boldsymbol{x}) = \sqrt{\frac{\sigma(x')}{\sigma(x)}} \tag{8}$$

$$\boldsymbol{Complexity}(\boldsymbol{x}) = \frac{\mathrm{Mobility}(\mathrm{x}')}{\mathrm{Mobility}(\mathrm{x})} \tag{9}$$

where μ_x represents the mean of x, x' represents the derivative of x, and $\sigma(x)$ represents the standard deviation of x. The activity parameter was equal to the variance.

1.5 Lyapunov Exponent (LE) [5]

The LE indicates the deterministic chaos of dynamics for time series produced by dynamic systems. The formula for the calculation of LE (λ) is:

$$\lambda(i) = \frac{1}{i\Delta t}\frac{1}{K}\sum_{\mathrm{j}=1}^{K} \ln \frac{d_j(i)}{d_j(0)} \tag{10}$$

where:

- i is the discrete time step,
- Δt is the sampling period of the EEG time series,
- K is associated with the embedding dimension set to 5 in this study,
- $d_j(0)$ is the initial distance from the jth point to its nearest neighbor, and
- $d_j(i)$ is the distance between the jth pair of nearest neighbors after i discrete time steps.

1.6 Hurst Exponent (HE) [6]

The HE has been widely used to evaluate the self-similarity and correlation properties of fractal Brownian noise, which is the time series produced by a fractional Gaussian process. HE is defined as follows:

$$HE = \log(R/S)/\log(N) \tag{11}$$

where N is the length of the data sample, R is the difference between the maximum deviation from the mean and the minimum deviation from the mean, and S is the standard deviation.

1.7 Kolmogorov Complexity (KC) [7]

The KC is a non-linear measure that is used to quantify the complexity or irregularity of a signal. To calculate KC, the EEG data are converted to a binary sequence having the same length, whose data value is 0 if it is less than the mean and 1 if it is larger. The sequence is used to calculate the number $c(n)$, which reflects the relative complexity of the data [8]. Then, the KC is

$$KC = c(n)/b(n) \tag{12}$$

where n is the length of the time-series data and $b(n)$ is the ratio between n and $log(n)$.

Supplementary Table 1. Performances of the top 10 electrode configurations composed of two electrodes. The table lists the calculated accuracies, sensitivities, and specificities for the top 10 electrode configurations with the highest accuracies. These values were used as performance measures.

Channel combination	Accuracy	Sensitivity	Specificity
**F3-F4**	74.06%	63.95%	84.13%
**CP5-CP6**	72.00%	67.35%	76.64%
**O1-O2**	70.98%	99.55%	42.40%
**FC5-FC6**	70.18%	52.38%	87.98%
**C3-C4**	69.16%	74.60%	63.72%
**T7-T8**	66.55%	67.12%	65.99%
**CP1-CP2**	65.53%	58.73%	72.34%
**AF3-AF4**	64.63%	57.14%	72.11%
**P3-P4**	63.61%	79.14%	48.07%
**Cz-Pz**	62.02%	44.90%	79.14%

Supplementary Table 2. Performances of the top 10 electrode configurations composed of four electrodes. The table lists the calculated accuracies, sensitivities, and specificities for the top 10 electrode configurations with the highest accuracies. These values were used as performance measures.

Channel combination	Accuracy	Sensitivity	Specificity
**AF3-AF4-FC5-FC6**	82.43 %	70.75%	94.10%
**F3-F4-FC5-FC6**	79.93%	78.91%	80.95%
**F3-F4-C3-C4**	76.19%	73.02%	79.37%
**AF3-AF4-F3-F4**	76.19%	70.07%	82.31%
**C3-C4-CP5-CP6**	75.96%	69.61%	82.31%
**P3-P4-O1-O2**	75.62%	81.63%	69.61%
**C3-C4-O1-O2**	74.94%	80.27%	69.61%
**FC5-FC6-C3-C4**	73.92%	66.68%	81.18%
**F3-F4-FC1-FC2**	73.47%	63.49%	83.90%
**Cz-Oz-F3-F4**	73.24%	62.81%	83.67%

Supplementary Table 3. Performances of the top 20 electrode configurations composed of six electrodes. The table lists the calculated accuracies, sensitivities, and specificities for the top 20 electrode configurations with the highest accuracies. These values were used as performance measures.

Channel combination	Accuracy	Sensitivity	Specificity
**FC5-FC6-C3-C4-P7-P8**	86.28 %	86.17 %	86.39%
**F3-F4-FC5-FC-P7-P8**	85.26%	83.22%	87.30%
**AF3-AF4-F3-F4-FC5-FC6**	84.47%	79.82%	89.12%
**Fz-Cz-AF3-AF4-FC5-FC6**	82.43%	70.75%	94.10%
**Fz-Pz-AF3-AF4-FC5-FC6**	82.43%	70.75%	94.10%
**Fz-Oz-AF3-AF4-FC5-FC6**	82.43%	70.75%	94.10%
**Cz-Pz-AF3-AF4-FC5-FC6**	82.43%	70.75%	94.10%
**Cz-Oz-AF3-AF4-FC5-FC6**	82.43%	70.75%	94.10%
**Pz-Oz-AF3-AF4-FC5-FC6**	82.43%	70.75%	94.10%
**AF3-AF4-FC5-FC6-FC1-FC2**	82.43%	70.75%	94.10%
**AF3-AF4-FC5-FC6-PO3-PO4**	82.20%	70.52%	93.88%
**AF3-AF4-FC5-FC6-CP1-CP2**	81.97%	70.52%	93.42%
**Fp1-Fp2-AF3-AF4-FC5-FC6**	81.86%	79.82%	92.97%
**AF3-AF4 -F7-F8-FC5-FC6**	81.86%	70.75%	93.42%
**AF3-AF4-FC5-FC6-C3-C4**	81.18%	70.75%	92.97%
**AF3-AF4-FC5-FC6-P7-P8**	81.07%	70.75%	93.42%
**Fp1-Fp2-FC5-FC6-P7-P8**	80.16%	70.75%	80.95%
**F3-F4-FC5-FC6-P3-P4**	79.82%	70.75%	79.14%
**FC5-FC6-CP5-CP6-P7-P8**	79.48%	70.75%	82.09%
**AF3-AF4-FC5-FC6-T7-T8**	79.37%	70.75%	89.57%

Supplementary Table 4. Performances of the top 20 electrode configurations composed of eight electrodes. The table lists the calculated accuracies, sensitivities, and specificities for the top 20 electrode configurations with the highest accuracies. These values were used as performance measures.

Channel combination	Accuracy	Sensitivity	Specificity
**F3-F4-FC5-FC6-C3-C4-P7-P8**	86.85 %	86.85%	86.85%
**FC5-FC6 -FC1-FC2-C3-C4-P7-P8**	85.83%	85.94%	85.71%
**Cz-Oz-FC5-FC6-C3-C4-P7-P8**	85.71%	85.71%	85.71%
**AF3-AF4-FC5-FC6-C3-C4-P7-P8**	85.49%	81.63%	89.34%
**Fz-Cz-FC5-FC6-C3-C4-P7-P8**	85.15%	85.49%	84.81%
**FC5-FC6-C3-C4-P7-P8-O1-O2**	85.03%	86.85%	83.22%
**F3-F4-FC5-FC6-FC1-FC2-P7-P8**	85.03%	83.45%	86.62%
**Fz-Oz-FC5-FC6-C3-C4-P7-P8**	84.81%	85.26%	84.35%
**Cz-Pz-FC5-FC6-C3-C4-P7-P8**	84.81%	84.13%	85.48%
**Fz-Cz-F3-F4-FC5-FC6-P7-P8**	84.69%	82.77%	86.62%
**Pz-Oz-FC5-FC6-C3-C4-P7-P8**	84.47%	83.90%	85.03%
**AF3-AF4-F3-F4-FC5-FC6-FC1-FC2**	84.35%	79.59%	89.12%
**Cz-Oz-F3-F4-FC5-FC6-P7-P8**	84.24%	82.54%	85.94%
**Fz-Cz-AF3-AF4-F3-F4-FC5-FC6**	84.24%	79.82%	88.66%
**Fz-Pz-FC5-FC6-C3-C4-P7-P8**	84.13%	83.90%	84.35%
**Cz-Pz-F3-F4-FC5-FC6-P7-P8**	84.13%	81.63%	86.62%
**Fp1-Fp2-AF3-AF4-F3-F4-FC5-FC6**	84.13%	80.27%	87.98%
**F7-F8-FC5-FC6-C3-C4-P7-P8**	84.01%	83.90%	84.13%
**FC5-FC6-C3-C4-CP1-CP2-P7-P8**	84.01%	82.77%	85.26%
**Cz-Pz-AF3-AF4-F3-F4-FC5-FC6**	84.01%	79.37%	88.66%

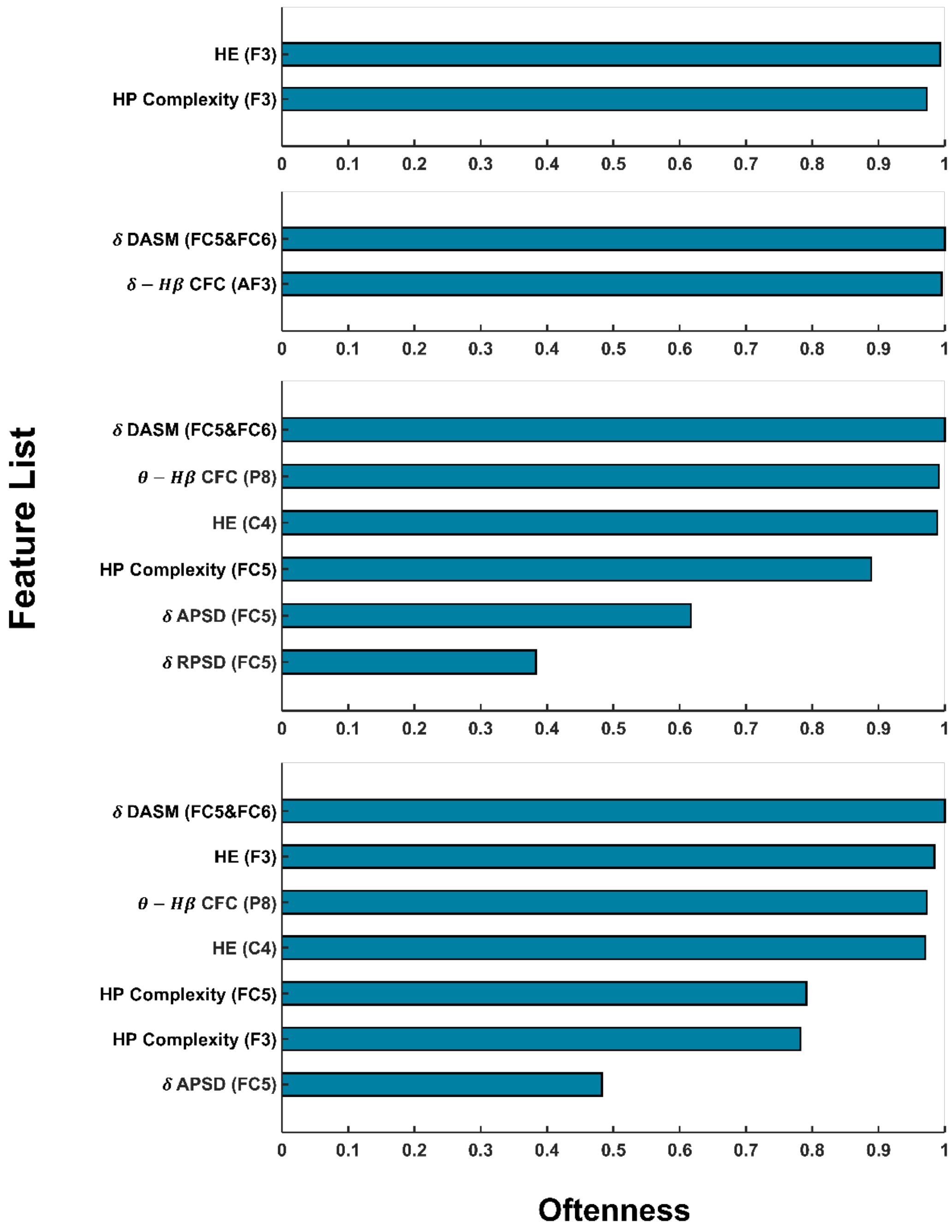

Supplementary Figure 1. List of the most frequently selected features. The features were most frequently selected in all CV iterations for the number of features that yielded the highest accuracy in each optimal electrode configuration. From top to bottom, the feature list of the Opt-2ch, Opt-4ch, Opt-6ch, and Opt-8ch configurations are described. The x-axis is "oftenness," which is calculated by dividing the feature selection times by all CV iteration times (441).

References

1. Osipova, D., D. Hermes, and O. Jensen, *Gamma power is phase-locked to posterior alpha activity.* PloS one, 2008. **3**(12): p. e3990.
2. Onslow, A.C., R. Bogacz, and M.W. Jones, *Quantifying phase–amplitude coupling in neuronal network oscillations.* Progress in biophysics and molecular biology, 2011. **105**(1-2): p. 49-57.
3. Shannon, C.E., *A mathematical theory of communication.* The Bell system technical journal, 1948. **27**(3): p. 379-423.
4. Hjorth, B., *The physical significance of time domain descriptors in EEG analysis.* Electroencephalography and clinical neurophysiology, 1973. **34**(3): p. 321-325.
5. Rosenstein, M.T., J.J. Collins, and C.J. De Luca, *A practical method for calculating largest Lyapunov exponents from small data sets.* Physica D: Nonlinear Phenomena, 1993. **65**(1-2): p. 117-134.
6. Feder, J., *Fractals*. 2013: Springer Science & Business Media.
7. Kolmogorov, A.N., *Three approaches to the definition of the concept "quantity of information".* Problemy peredachi informatsii, 1965. **1**(1): p. 3-11.
8. Lempel, A. and J. Ziv, *On the complexity of finite sequences.* IEEE Transactions on information theory, 1976. **22**(1): p. 75-81.
